# A culture collection of bacterial isolates from Lake Erie and Lake Victoria

**DOI:** 10.1128/mra.00558-26

**Published:** 2026-08-07

**Authors:** Jeremiah A. Adesanya, Megan P. Lynagh, Katelyn M. Brown, Christopher S. Ward

**Affiliations:** 1 Department of Biological Sciences, Bowling Green State University 1888, Bowling Green, Ohio, USA; 2 Department of Estuarine and Ocean Sciences, University of Massachusetts Dartmouth 145770, New Bedford, Massachusetts, USA; Indiana University Bloomington, Bloomington, Indiana, USA

**Keywords:** Great Lakes, bacterial isolation, culture collection

## Abstract

We present a culture collection of 44 aquatic bacterial cultures isolated from samples collected from Lake Erie’s Sandusky and Maumee Bays and Lake Victoria’s Winam Gulf. Here, we detail isolation, cryopreservation, and genomic DNA preparation methods and sequences of full-length 16S rRNA genes.

## ANNOUNCEMENT

Freshwater bacteria play crucial roles in biogeochemical transformations in eutrophic lake systems, particularly lacustuaries where riverine inputs mix with lake water, and bacterial remineralization can have significant impacts on phytoplankton growth (1, 2). Dynamics between bacterial and phytoplankton communities and how they influence the physicochemical nature of the lakes are critical factors in understanding the productivity and function of the Global Great Lakes freshwater ecosystems (3, 4). This culture collection will facilitate freshwater microbial ecology studies of both Great Lakes systems and aid validation of microbial metabolic contributions to global biogeochemical cycles.

**Fig 1:**
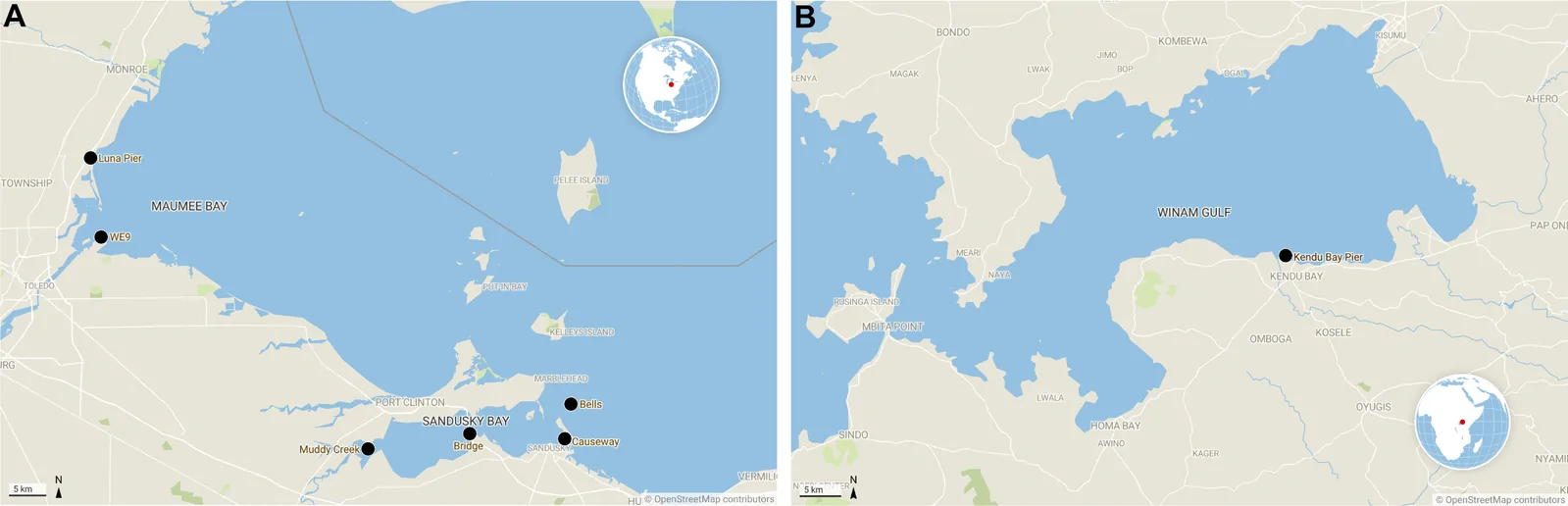
Sites of water collection for bacterial isolations. (**A**) Lake Erie, with sites in Maumee Bay (WE9, Luna Pier), Sandusky Bay (Muddy Creek, bridge, and causeway), and open lake (Bells). (**B**) Lake Victoria, with a site in Winam Gulf (Kendu Bay Pier). Maps created with Datawrapper.

Bacterial strains were isolated from several sites in Lake Erie and Lake Victoria (Fig. 1) between June and September 2023. Lake water was collected from research vessels using a Van Dorn sampler, transported on ice to the lab where it was stored at 4°C, and processed within 24 h. Water was plated on yeast mannitol agar with Congo Red (YMA + CR) and incubated at room temperature (23°C) until visible growth was evident. Isolation conditions and other information are included in Table 1. Distinct colonies were re-streaked on tryptic soy agar (TSA, BD Difco) for purification, and 700 µL of overnight cultures of each isolate was combined with 300 µL of 30% glycerol in cryovial tubes, then flash-frozen and stored at −80°C. To prepare cultures for DNA extraction, isolates were streaked on TSA and incubated for 24–48 h at 30°C, and colonies were transferred to tryptic soy broth and incubated by shaking at 200 rpm for 24–48 h at room temperature. One milliliter aliquots of each isolate culture were pelleted by centrifuging at 5,000 × *g* for 3 min, and genomic DNA was extracted using a DNeasy PowerWater Kit (QIAGEN, USA).

**TABLE 1:** Isolate ID, isolation conditions, and NCBI BLAST details for the aquatic bacterial isolates

Isolate ID	BLAST genus	BLAST species	Percentage identity (%)	BLAST Hit accession no.	Isolation date (month/day/year)	Isolation location	GenBank accession no.	SRA accession no.	BioSample accession no.
KMB_001	*Caulobacter*	sp.	99.17	MZ490613.1	5/29/2023	Winam Gulf: Kendu Bay Pier	PZ618271	SRR38023598	SAMN57184910
KMB_002	*Enterobacter*	*cloacae*	98.98	LC705407.1	5/29/2023	Winam Gulf: Kendu Bay Pier	PZ618272	SRR38104201	SAMN57247327
KMB_003	*Enterobacter*	*cloacae*	98.22	LC705407.1	5/29/2023	Winam Gulf: Kendu Bay Pier	PZ618273	SRR38104200	SAMN57247328
KMB_004	*Enterobacter*	*cloacae*	98.62	LC705407.1	5/29/2023	Winam Gulf: Kendu Bay Pier	PZ618274	SRR38104189	SAMN57247329
KMB_005	*Pseudomonas*	*putida*	99.58	JQ782496.1	8/29/2023	Sandusky Bay: causeway	PZ618275	SRR38104178	SAMN57247330
KMB_006	*Vogesella*	sp.	99.57	OQ217595.1	8/29/2023	Sandusky Bay: causeway	PZ618276	SRR38104175	SAMN57247331
KMB_007	*Vogesella*	sp.	96.69	OQ217595.1	8/29/2023	Sandusky Bay: causeway	PZ618277	SRR38104174	SAMN57247332
KMB_008	*Gemmobacter*	*lanyuensis*	97.92	NR_109450.1	8/28/2023	Lake Erie: Luna Pier	PZ618278	SRR38104173	SAMN57247333
KMB_009	*Gemmobacter*	*lanyuensis*	99.42	NR_109450.1	8/28/2023	Lake Erie: Luna Pier	PZ618279	SRR38104172	SAMN57247334
KMB_011	*Aeromonas*	sp.	99.18	ON203098.1	9/7/2023	Lake Erie: WE9	PZ618280	SRR38104171	SAMN57247335
MPL_001	*Enterobacter*	sp.	88.03	PP218373.1	6/7/2023	Sandusky Bay: Muddy Creek	PZ618281	SRR38104169	SAMN57247838
MPL_002	*Acinetobacter*	sp.	99.35	MK828175.1	6/7/2023	Sandusky Bay: Muddy Creek	PZ618282	SRR38104168	SAMN57247839
MPL_003	*Bacillus*	sp.	98.52	AB740353.1	6/7/2023	Sandusky Bay: Muddy Creek	PZ618283	SRR38104165	SAMN57247840
MPL_004	*Bacillus*	sp.	99.02	KX078248.1	6/7/2023	Sandusky Bay: Muddy Creek	PZ618284	SRR38104164	SAMN57247841
MPL_006	*Priestia*	*megaterium*	98.34	KC969078.1	6/7/2023	Sandusky Bay: Muddy Creek	PZ618285	SRR38104163	SAMN57247842
MPL_007	*Enterobacter*	sp.	99.60	DQ279307.1	6/7/2023	Sandusky Bay: bridge	PZ618286	SRR38104162	SAMN57247843
MPL_008	*Mesobacillus*	sp.	96.67	NR_163641.1	6/7/2023	Sandusky Bay: bridge	PZ618287	SRR38104161	SAMN57247844
MPL_009	*Flavobacterium*	sp.	97.88	JX290486.1	6/7/2023	Sandusky Bay: bridge	PZ618288	SRR38104160	SAMN57247845
MPL_010	*Bacillus*	sp.	98.97	OQ719226.1	6/7/2023	Sandusky Bay: bridge	PZ618289	SRR38104159	SAMN57247846
MPL_011	*Flavobacterium*	sp.	98.58	JX657115.1	6/7/2023	Sandusky Bay: bridge	PZ618290	SRR38104158	SAMN57247847
MPL_013	*Vogesella*	sp.	99.78	MG818314.1	6/7/2023	Sandusky Bay: bridge	PZ618291	SRR38104166	SAMN57247849
MPL_014	*Sphingobium*	*xenophagum*	100.00	MF093198.1	6/29/2023	Lake Erie: Bells	PZ618292	SRR38104170	SAMN57247336
MPL_015	*Sphingobium*	*xenophagum*	99.38	KY357346.1	6/29/2023	Lake Erie: Bells	PZ618293	SRR38104199	SAMN57247337
MPL_016	*Nocardioides*	*kribbensis*	99.49	NR_043215.1	6/29/2023	Lake Erie: Bells	PZ618294	SRR38104198	SAMN57247338
MPL_017	*Rheinheimera*	sp.	99.40	KF556693.1	6/29/2023	Lake Erie: Bells	PZ618295	SRR38104197	SAMN57247339
MPL_018	*Nocardioides*	*kribbensis*	94.82	OQ221835.1	6/29/2023	Lake Erie: Bells	PZ618296	SRR38104196	SAMN57247340
MPL_019	*Brevundimonas*	sp.	99.50	HQ436453.1	6/29/2023	Lake Erie: Bells	PZ618297	SRR38104195	SAMN57247341
MPL_020	*Brevundimonas*	*aurantiaca*	99.87	HQ436453.1	6/29/2023	Lake Erie: Bells	PZ618298	SRR38104194	SAMN57247342
MPL_022	*Stenotrophomonas*	*maltophilia*	99.70	PQ856812.1	6/29/2023	Lake Erie: Bells	PZ618299	SRR38104193	SAMN57247343
MPL_023	*Micrococcus*	sp.	99.69	JN866765.1	6/29/2023	Lake Erie: Bells	PZ618300	SRR38104192	SAMN57247344
MPL_024	*Rheinheimera*	sp.	99.12	KT826393.1	6/29/2023	Lake Erie: Bells	PZ618301	SRR38104191	SAMN57247345
MPL_025	*Brevundimonas*	sp.	98.82	HQ436453.1	6/29/2023	Lake Erie: Bells	PZ618302	SRR38104190	SAMN57247346
MPL_026	*Caulobacter*	sp.	98.63	AJ227764.1	6/29/2023	Lake Erie: Bells	PZ618303	SRR38104188	SAMN57247347
MPL_027	*Bacillus*	*subtilis*	99.39	OR975488.1	6/29/2023	Lake Erie: Bells	PZ618304	SRR38104187	SAMN57247348
MPL_028	*Chryseobacterium*	sp.	99.19	GU296676.1	6/29/2023	Lake Erie: Bells	PZ618305	SRR38104186	SAMN57247349
MPL_029	*Rhizobium*	sp.	99.09	MK584885.1	6/29/2023	Sandusky Bay: Muddy Cree	PZ618306	SRR38104185	SAMN57247350
MPL_030	*Rhizobium*	sp.	100	MK584885.1	6/29/2023	Sandusky Bay: Muddy Creek	PZ618307	SRR38104184	SAMN57247351
MPL_031	*Bacillus*	sp.	98.16	ON247992.1	6/29/2023	Sandusky Bay: Muddy Creek	PZ618308	SRR38104183	SAMN57247352
MPL_032	*Bacillus*	sp.	98.57	MH107110.1	6/29/2023	Sandusky Bay: Muddy Creek	PZ618309	SRR38104182	SAMN57247353
MPL_033	*Aeromonas*	*veronii*	97.73	PQ119096.1	6/29/2023	Sandusky Bay: Muddy Creek	PZ618310	SRR38104181	SAMN57247354
MPL_034	*Bacillus*	sp.	98.23	KY625514.1	6/29/2023	Sandusky Bay: Muddy Creek	PZ618311	SRR38104180	SAMN57247355
MPL_035	*Bacillus*	sp.	97.69	PV055963.1	6/29/2023	Sandusky Bay: Muddy Creek	PZ618312	SRR38104179	SAMN57247356
MPL_036	*Bacillus*	sp.	98.71	MW822752.1	6/29/2023	Sandusky Bay: Muddy Creek	PZ618313.	SRR38104177	SAMN57247357
MPL_037	*Delftia*	*acidovorans*	99.38	PP389992.1	6/29/2023	Sandusky Bay: Muddy Creek	PZ618314	SRR38104176	SAMN57247358

To identify the taxonomic and phylogenetic relatedness of each isolate, PCR was performed to amplify full-length 16S rRNA genes. PCR Master Mix (25 µL; cat. no. K0171; Thermo Scientific, USA) containing 0.05 U/µL Taq DNA polymerase, reaction buffer, 4 mM MgCl_2_ and 0.4 mM of dNTP was combined with 1.0 µM universal bacterial primers 27F (5′-AGAGTTTGATCMTGGCTCAG-3′) and 1492R (5′-CGGTTACCTTGTTACGACTT-3′) (5; Integrated DNA Technologies, USA), 1 µL DNA templates, and 22 µL nuclease-free water. The thermocycler program was 95°C for 5 min for the initial denaturation, 35 cycles of 72°C for 60 s, annealing at 65°C for 30 s, extension at 72°C for 60 s, and final extension at 72°C for 5 min. Amplicon cleanup was done using ExoSAP-IT (Applied Biosystems, USA), and the quality was assessed by Nanodrop One Microvolume UV-Vis Spectrophotometer and gel electrophoresis. Sanger sequencing was performed by GENEWIZ (South Plainfield, NJ, USA). Ambiguous nucleotides were manually trimmed at the 5′ and 3′ ends of the sequences. Taxonomy was assigned by NCBI BLAST match using Megablast and excluding uncultured/environmental sample sequences.

The culture collection includes isolates belonging to 20 unique genera, across Actinobacteria, Bacteroidetes, Firmicutes, and Proteobacteria phyla. *Caulobacter* and *Enterobacter* isolates were collected from both Lake Erie and Lake Victoria.

## Data Availability

Full-length 16S rRNA consensus sequences have been deposited to the NCBI SRA database, and raw reads are available in the NCBI SRA database under BioProject accession number PRJNA1451202 and GenBank accession number PZ618271–PZ618314. The bacterial isolates are available upon request from Christopher Ward (cward8@umassd.edu).
